# Spectrum of Ixodidae Ticks Attacking Humans in Novosibirsk Province, Russian Siberia, and Their Association with Tick-Borne Bacterial Agents

**DOI:** 10.3390/pathogens14040315

**Published:** 2025-03-25

**Authors:** Vera Rar, Galina Chicherina, Yana Igolkina, Valeria Fedorets, Tamara Epikhina, Nina Tikunova

**Affiliations:** 1 Institute of Chemical Biology and Fundamental Medicine SB RAS, 630090 Novosibirsk, Russia; igolkina@inbox.ru (Y.I.); v.fedorets@g.nsu.ru (V.F.); tikunova@niboch.nsc.ru (N.T.); 2 Institute of Systematics and Ecology of Animals SB RAS, 630091 Novosibirsk, Russia; chicherinagalina@bk.ru

**Keywords:** *Ixodes pavlovskyi*, *Ixodes persulcatus*, *Dermacentor reticulatus*, engorged ticks, human blood, *Borrelia burgdorferi* sensu lato (s.l.), *Borrelia miyamotoi*, *Rickettsia* spp., Anaplasmataceae

## Abstract

The spectrum of ixodid ticks that bite humans in Western Siberia has significantly changed over the past two decades. In this study, we determined tick species attacking people in the vicinity of Novosibirsk and the range of bacterial agents they were infected with. This study included 301 ticks taken from people and 46% were *Ixodes pavlovskyi*, followed by *Ixodes persulcatus* (19.6%), *I. persulcatus*/*I. pavlovskyi* interspecies hybrids (19.6%), *Dermacentor reticulatus* (12.8%), and single *Dermacentor marginatus* and *Dermacentor nuttalli*/*Dermacentor silvarum*. Human DNA was determined in ticks, first demonstrating that all *Ixodes* spp., including hybrids, can effectively feed on humans. The DNA of *Borrelia* spp., *Rickettsia* spp., and Anaplasmataceae bacteria was detected in different tick species. *Borrelia garinii* prevailed in *Ixodes* species, being found in 8.8% of ticks, whereas *B. afzelii* and *B. bavariensis* were found in single ticks. *Borrelia miyamotoi* was revealed in 3.7% of ticks. “*Candidatus* Rickettsia tarasevichiae” and *R. raoultii* were identified mainly in *I. persulcatus* and *D. reticulatus* (44.8% and 26.3%, respectively), while *Rickettsia helvetica* was found only in 2.2% *I. pavlovskyi*. The prevalence of *Anaplasma phagocytophilum*, *Ehrlichia muris,* and *Neoehrlichia mikurensis* did not exceed 2%. The obtained results indicate a high risk for humans to be infected with agents of Lyme borreliosis, primarily *B. garinii*.

## 1. Introduction

Several species of ixodid ticks inhabit Novosibirsk province, which is located in the southern part of Russian Western Siberia. Among ticks that bite humans, the most prevalent are *Ixodes persulcatus*, *Ixodes pavlovskyi*, and *Dermacentor reticulatus*, while *Dermacentor marginatus* and *Dermacentor silvarum* are less common [1,2].

*Ixodes persulcatus* exhibits a wide distribution area in the forest regions of Russia, extending from the North-Western to the Far Eastern regions. Until the late 20th century, *I. persulcatus* was the predominant tick species in all examined locations of the Siberian forest zone. In contrast to *I. persulcatus*, *I. pavlovskyi* displays a discontinuous distribution, inhabiting the Far Eastern and Western Siberian regions [2,3,4,5,6]. In the 21st century, interest in the study of *I. pavlovskyi* has increased sharply due to the rapid and significant spread of this tick. Having previously been primarily confined to the mountainous regions of Siberia (Altai, Kuznetsk Alatau, and Salair Ridge) [4,7,8], the habitat of *I. pavlovskyi* has expanded to include not only foothills but also lowland biotopes, especially around major cities such as Novosibirsk and Tomsk [1,9,10]. Furthermore, the presence of natural interspecies hybrids of *I. persulcatus*/*I. pavlovskyi* (hereinafter referred to as hybrids) has been demonstrated in all examined locations where these two species are known to coexist, namely in the Republic of Altai and Novosibirsk and Tomsk provinces in Siberia as well as in Russky Island in the Far East [11,12,13]. Both these *Ixodes* species and their hybrids are infected with the same tick-borne agents, including highly pathogenic tick-borne encephalitis virus, agents of Lyme borreliosis (LB) and *Borrelia miyamotoi* disease (BMD), as well as several *Rickettsia* species and bacteria from the Anaplasmataceae family [10,12,14,15,16,17]. Notably, the prevalence of some agents varied depending on the tick species. Thus, *I. persulcatus* was significantly more often infected with *Borrelia bavariensis* and “*Candidatus* Rickettsia tarasevichiae” and less often with *Borrelia garinii* compared to *I. pavlovskyi* [10].

Unlike *Ixodes* spp., *Dermacentor* spp. ticks carry mainly rickettsial pathogens; of them, *Rickettsia raoultii* is frequently found, while *Rickettsia sibirica*, the highly pathogenic causative agent of Siberian tick typhus (STT), is rarely detected [18,19,20,21,22]. Compared to *Ixodes* spp., *D. reticulatus,* and *D. marginatus* inhabit drier and warmer areas located in forest–steppe and steppe zones, whereas *D. silvarum* inhabits forest areas; in lowland locations of Western Siberia, the number of *D. silvarum* is low [2].

Despite the distribution of different Ixodidae ticks in Western Siberia being examined in a number of studies [1,9,23,24], the epidemiological significance of *I. pavlovskyi* and *I. persulcatus*/*I. pavlovskyi* interspecies hybrids recently invaded this region has not been sufficiently studied. To clarify this issue, in this study, we examined the spectrum of ticks attacking humans in Novosibirsk province (Western Siberia), and their association with various bacterial agents.

## 2. Materials and Methods

### 2.1. Sampling

**Figure 1:**
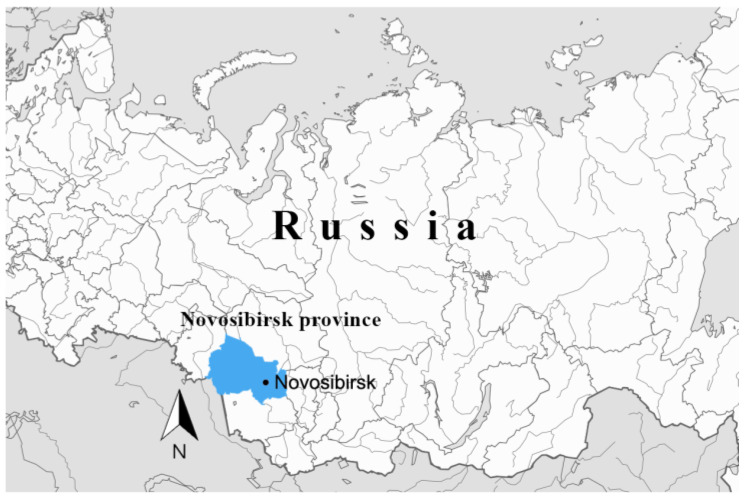
The map shows the location of tick collection.

The study included ixodid ticks that attacked people. A total of 301 ticks were collected from 300 residents of Novosibirsk in period of maximal activity of *Ixodes* spp. (May–June) in 2021–2024 (Figure 1). Attached and non-attached ticks were removed by laboratory clients from their bodies or clothing and submitted to the laboratory. This study did not analyze information on the gender, age, occupation, and health status of people who submitted ticks, or the locations where ticks attacked people.

### 2.2. DNA Extraction and Quantification

Total DNA was extracted from ticks using “Real Best Extraction 100” kit (“Vector-Best”, Novosibirsk, Russian Federation), according to the protocol, and 50 µL from 400 µL of extracted DNA from each tick was used for this study. DNA concentration was measured using a Qubit dsDNA HS kit (Life Technology, Carsbad, CA, USA). Depending on tick species and degree of engorgement, DNA concentration varied from 0.8 to 7.2 ng/µL.

### 2.3. Tick Species Determination

The tick species was determined based on the results of species-specific PCR of the mitochondrial cytochrome c oxidase subunit 1 (*cox*1) gene and sequencing of a fragment of the nuclear multi-copy internal transcribed spacer (ITS2). For all samples, *cox*1 gene fragments were amplified using primers specific to *I. persulcatus*, *I. pavlovskyi,* and *D. reticulatus*. For subsequent sequencing, ITS2 fragments were amplified for all *Ixodes* spp. and a number of *Dermacentor* spp. ticks using primers indicated in Table 1. Ticks with ITS2 fragments, which were heterozygous at characteristic positions that differ among *I. persulcatus* and *I. pavlovskyi*, were termed ticks with hybrid ITS2 fragments. *Ixodes* spp. ticks with mitochondrial and nuclear loci belonging to the same species were identified as *I. persulcatus* or *I. pavlovskyi*. The determination of hybrids was carried out as previously described [12]. *Ixodes* spp. ticks with hybrid ITS2 fragments and ticks with mitochondrial and nuclear loci belonging to different species were defined as hybrids.

Ticks with *cox*1 fragments corresponding to *D. reticulatus* were identified as *D. reticulatus*, whereas the species of other *Dermacentor* spp. were determined based on sequencing of ITS2 and *cox*1 gene fragments using primers specified in Table 1.

### 2.4. Detection of Human DNA in Ticks as a Proxy for Engorgement

To identify the engorged ticks and estimate the degree of engorgement, all ticks were examined for the presence of human DNA by RT-PCR with TaqMan probe targeted to the TPOX locus, human tyrosine hydroxylase gene, as previously described [25]. To standardize this assay, DNA isolated from 100 µL of human blood and serially diluted 10-fold was used as positive control. The results were considered positive if cycle threshold (Ct) was <40.

### 2.5. Detection and Genetic Characterization of Bacterial Agents

Identification of bacterial agents in tick specimens was carried out by genus-specific and species-specific PCR using primers specified in Table 1 and/or subsequent sequencing, as previously described [10].

*Borrelia burgdorferi* sensu lato (s.l.) and *B. miyamotoi* DNA was detected using multiplex PCR targeted to the 5S-23S rRNA intergenic spacer (IGS) of *B. burgdorferi* s.l. and the *p66* gene of *B. miyamotoi*. For positive *B. burgdorferi* s.l. specimens, additional PCR assays with primers specific to *clp*A and *p83*/*100* genes were carried out and the obtained PCR fragments were sequenced (Table 1). To determine *B. burgdorferi* genospecies, *clp*A gene sequences were analyzed using Public Databases for Molecular Typing and Microbial Genome Diversity (PubMLST; https://pubmlst.org/organisms/borrelia-spp), accessed on 20 November 2024, and Blastn (https://blast.ncbi.nlm.nih.gov) of National Center for Biotechnology Information (NCBI), accessed on 20 November 2024. To determine the genospecies of samples that could not be amplified by the *clp*A gene, the obtained *p83*/*100* gene or IGS sequences were compared with available sequences using Blastn search.

**Table 1:** Primers used for identification of tick species and bacterial agents.

Locus	Organism	Reaction	Primer Name	Primer Sequences 5′-3′	T* (°C)	References
ITS2	Ixodidae	conventional	F-ITS2	acacactgagcacttactctttga	55	[26]
			R-ITS800	gggggttgtctcgcctgatgt		
*cox*1	*I. persulcatus*	Conventional	Ixodes-F	acctgatatagctttccctcg	55	[10]
			Ipers-R	ttgattcctgttggaacagc		
	*I. pavlovskyi*	Conventional	Ixodes-F	acctgatatagctttccctcg	55	[10]
			Ipav-R	taatccccgtggggacg		
	Ixodidae	Conventional	C1	accacaaagacattggaactatatat	50	[23]
			C2	aatccaggaagaataagaatatatac		
	*D. reticulatus*	Conventional	Dret-F	ctaagacaacccggaacattaattg	60	This study
			Dret-R	aaaccctaaaagaccaattgcggc		
IGS	*B. burgdorferi* s.l.	Primary	NC1	cctgttatcattccgaacacag	50	[10]
			NC2	tactccattcggtaatcttggg		
		Nested	NC3	tactgcgagttcgcgggag	50	
			NC4	cctaggcattcaccatagac		
*p66*	*B. miyamotoi*	Primary	M3	ttctatatttggacacatgtc	50	[27]
			M4	cagattgtttagttctaatccg		
		Nested	M1	ctaaattattaaatccaaaatcg	50	
			M2	ggaaatgagtacctacatatg		
*clpA*	*B. burgdorferi* s.l.	Primary	clpAF1237	aaagatagatttcttccagac	50	[28]
			clpAR2218	gaatttcatctattaaaagctttc		
		Nested	clpAF1255	gacaaagcttttgatattttag	50	
			clpAR2104	caaaaaaaacatcaaattttctatctc		
*p83*/*100*	*B. burgdorferi* s.l.	Primary	F7	ttcaaagggatactgttagagag	50	[10]
			F10	aagaaggcttatctaatggtgatg		
		Nested	F5	acctggtgatgtaagttctcc	54	
			F12	ctaacctcattgttgttagactt		
*gltA*	*Rickettsia* spp.	Primary	glt1	gattgctttacttacgaccc	52	[10]
			glt2	tgcatttctttccattgtgc		
		Nested	glt3	tatagacggtgataaaggaatc	53	
			glt4	cagaactaccgatttctttaagc		
	Ca. R. tarasevichiae	Nested	RT1	tactaaaaaagtcgctgttcattc	56	[10]
			RT2	tgttgcaaacatcatgcgtaa		
	SFGR	Nested	RH1	gtcagtctactatcacctatatag	54	[10]
			RH3	taaaatattcatctttaagagcga		
*ompB*	*Rickettsia* spp.	Primary	B1	atatgcaggtatcggtact	56	[29]
			B2	ccatataccgtaagctacat		
		Nested	B3	gcaggtatcggtactataaac	56	
			B4	aatttacgaaacgattacttccgg		
16S rRNA	Anaplasmataceae	Primary	Ehr1	gaacgaacgctggcggcaagc	57	[10]
			Ehr2	agtaycgraccagatagccgc		
		Nested	Ehr3	tgcataggaatctacctagtag	60	
			Ehr4	ctaggaattccgctatcctct		

T*—annealing temperature.

*Rickettsia* spp. was identified in tick samples using nested PCR with primers targeting the *glt*A and *omp*B genes, as described previously [10]. To determine possible mixed infection, all positive samples were independently amplified using primers RT1 and RT2 specific to “*Candidatus* R. tarasevichiae”, and primers RH1 and RH3 specific to spotted fever group rickettsiae (SFGR) (Table 1). The species of all SFGR were determined by sequencing of *glt*A or *omp*B gene fragments. For *R. helvetica*-positive samples, the sequences of a long fragment of the *omp*B gene with a total length of 3117 bp were determined, as previously described [30].

Anaplasmataceae bacteria DNA was revealed by nested PCR using primers targeted to 16S rRNA gene (Table 1). For species determination, the obtained PCR fragments were sequenced.

### 2.6. Sequencing and Phylogenetic Analysis

The obtained amplicons were gel purified in 0.6% SeaKem^®^ GTG-agarose (Lonza, Haifa, Israel). Sanger sequencing was carried out using BigDye Terminator V. 3.1 Cycling Sequencing Kit (Applied Biosystems, Carlsbad, CA, USA). Sanger reaction products were analyzed using an ABI 3500 Genetic Analyzer (Applied Biosystems Inc.).

Obtained sequences were compared with those of reference strains available on the NCBI website using BLASTN, accessed on 20 November 2024, and aligned using the software MEGA 7.0 (www.megasoftware.net). Phylogenetic analyses were performed with the maximum likelihood (ML) method. The best-fitting substitution model was determined with the Bayesian Information Criterion (BIC) using the ML model test implemented in MEGA 7.0 [31]. The sequences from the GenBank database used for phylogenetic analyses were selected based on (i) genetic similarity to the obtained sequences; (ii) fragment length; (iii) belonging to type strains. In each phylogenetic tree, a sequence most closely related to the sequences from a group of interest was used as an outgroup. All phylogenetic trees were midpoint-rooted.

### 2.7. Statistical Analysis

The Pearson’s *χ*^2^-test (https://www.socscistatistics.com/tests/chisquare2/default2.aspx (accessed on 20 November 2024)) was used for comparison of the portion of engorged ticks and prevalence of infectious agents in different tick species. If a statistically significant difference was detected using the *χ*^2^ test, the strength of the association between pathogens and tick species was approved using the Cramer V test. The significance of differences in the degree of engorgement between tick species was assessed using non-parametric Kruskal–Wallis H test in Python 3.12.4 (pandas 1.5.3, statannote 0.2.3). Mann–Whitney U test was used for further comparison of blood amounts between tick species pairs (https://www.socscistatistics.com/tests/mannwhitney/default2.aspx (accessed on 20 November 2024)) when significant differences were shown by Kruskal–Wallis H test. In all tests, *p* < 0.05 was considered as significant. The results of statistical tests were presented in APA style (https://apastyle.apa.org/ (accessed on 20 November 2024)).

### 2.8. Nucleotide Sequence Accession Numbers

Nucleotide sequences determined in the study are available in the GenBank database under accession numbers: PQ685972–PQ685977, PQ682399–PQ682400 and PV362830-PV362834 for *Dermacentor* spp.; PQ682653–PQ682655 for *B. miyamotoi*; PQ724397–PQ724411 for *B. burgdorferi* s.l.; PQ682631–PQ682652 for *Rickettsia* spp.

## 3. Results

### 3.1. Tick Species

A total of 301 specimens of ticks attacking humans in the vicinity of Novosibirsk were examined. Tick species were determined based on analysis of the mitochondrial *cox*1 gene and nuclear ITS2. We failed to determine tick species in five specimens; these samples were excluded, and 296 specimens were studied. Because of the probable presence of *I. persulcatus*/*I. pavlovskyi* interspecies hybrids among examined ticks, all *Ixodes* spp. were genetically characterized by both mitochondrial and nuclear loci, as previously described [12]. A total of 137 *I. pavlovskyi*, 58 *I. persulcatus*, and 58 hybrids were identified. Among the detected hybrids, 46 ticks contained hybrid ITS2 fragments. These hybrid variants may be the result of the crossing of *I. persulcatus* and *I. pavlovskyi* ticks and, therefore, correspond to the genotypes of F1 progeny. In addition, 12 hybrids contained mitochondrial and nuclear loci belonging to different species; these variants could have resulted from repeated crosses of hybrids with the parental species and thus correspond to F2 progeny.

**Figure 2:**
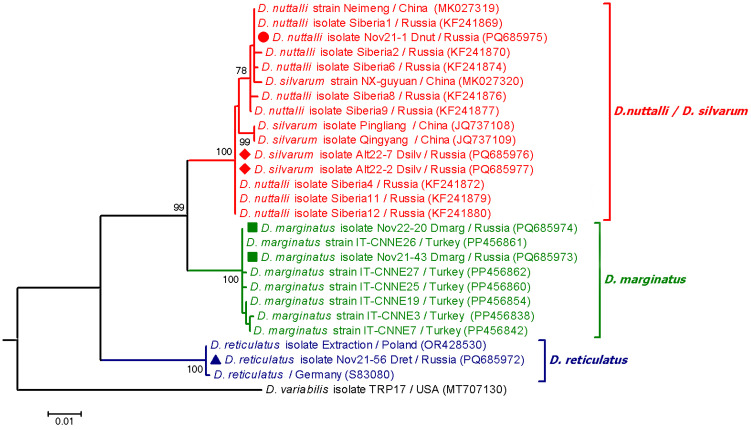
Phylogenetic tree constructed by the ML method (substitution model: Tamura 3-parameter with Gamma distribution (T92 + G)) based on nucleotide sequences of 1078 bp fragment of ITS2 of *Dermacentor* spp. The scale bar indicates an evolutionary distance of 0.01 nucleotide per position in the sequence. Significant bootstrapping values (>70%) are shown on the nodes. *Dermacentor variabilis* was used as an outgroup. Legend: ●—*Dermacentor nuttalli*; ♦—*Dermacentor silvarum*; ■—*Dermacentor marginatus*; ▲—*Dermacentor reticulatus*.

*Dermacentor reticulatus* DNA was found in 38 specimens based on PCR using species-specific primers by the *cox*1 gene. For 13 specimens, ITS2 or *cox*1 fragments of *D. reticulatus* were sequenced to confirm the correctness of *D. reticulatus* identification using primers designed in this study. All determined ITS2 fragments were identical and had four polymorphic sites in positions, which differentiate *D. reticulatus* haplotypes previously identified in Eurasia (Figure 2) [32]. The determined *cox*1 sequences of *D. reticulatus* were identical to those previously identified in Novosibirsk province (OM867332) or differed from this sequence by one unique nucleotide substitution.

Based on sequences of *cox*1 gene and ITS2 fragment, two *D. marginatus* and three *D. nuttalli*/*D. silvarum* were identified among the collected ticks. Two determined ITS2 sequences of *D. marginatus* had five and six polymorphic sites; the location of these sites differed in these sequences. The probable haplotypes of *D. marginatus* corresponded to those of *D. marginatus* from Turkey (PP456862, etc.) (Figure 2). Notably, this is the first determination of ITS2 sequences of *D. marginatus* collected in Russia. The determined *cox*1 gene sequences of *D. marginatus* were identical to each other and to the corresponding sequences of *D. marginatus* from Kazakhstan (MN907825) and China (NC_062069).

The ITS2 sequences of three *Dermacentor* spp. were closely related to available sequences of *D. nuttalli* and *D. silvarum* from Russian Siberia and China. Due to the high genetic similarity between *D. nuttalli* and *D. silvarum*, these tick species can be distinguished by morphology and distribution area rather than genetically. The determined in this study *D. nuttalli*/*D. silvarum* sequences of ITS2 fragment differed between themselves by eight mismatches (seven substitutions and one indel) and seven polymorphic sites. For two *D. nuttalli*/*D. silvarum* ticks, the ITS2 sequences (1069 bp fragments) were identical and showed the most similarity with *D. nuttalli* from the Baikal region (KF241872), differing from it by the presence of four polymorphic sites (Figure 2). However, since the locations where these ticks were taken are known and correspond to the distribution area of *D. silvarum*, but not *D. nuttalli* (forest biotopes), these ticks are probably *D. silvarum*. The ITS2 sequence of the third tick was the most similar to those of *D. nuttalli* from Baikal region (KF241869) and China (OQ955291), differing from them by three polymorphic sites (Figure 2). The obtained *cox*1 gene sequences of *D. nuttalli*/*D. silvarum* differed between themselves by seven nucleotide substitutions (98.9% similarity). The sequences of the two ticks were identical to each other and showed 100% similarity with the corresponding sequences of *D. silvarum* from China (MK028676), whereas the sequence of the third tick showed the highest similarity (99.7%) with *D. nuttalli* from China (KU594270).

### 3.2. Determination of Human DNA in Ticks

To determine the proportion of engorged ticks and to assess the degree of their engorgement, all specimens were tested for the presence of human DNA by TaqMan real-time PCR targeted to the human TPOX locus. The amount of human DNA corresponded to the volume of human blood. Experiments performed with serial dilutions of control human DNA showed a linear dependence between the dilution degree and the threshold cycle (Ct) up to Ct value below 42 (Supplementary Material, Figure S1); the results were considered positive if Ct was <40. An exponential increase in fluorescence channel was observed for both control human DNA in serial dilutions and positive tick specimens (Supplementary Material, Figure S2).

Human DNA was found in 72/296 (24.3%) tested specimens. Among PCR-positive ticks, 55/72 (76.4%) ticks contained a small amount of human DNA, equivalent to 0.4–5.0 µL of human blood, and 17/72 (22.6%) ticks contained human DNA, corresponding to 5–106 µL of blood (Table 2).

**Figure 3:**
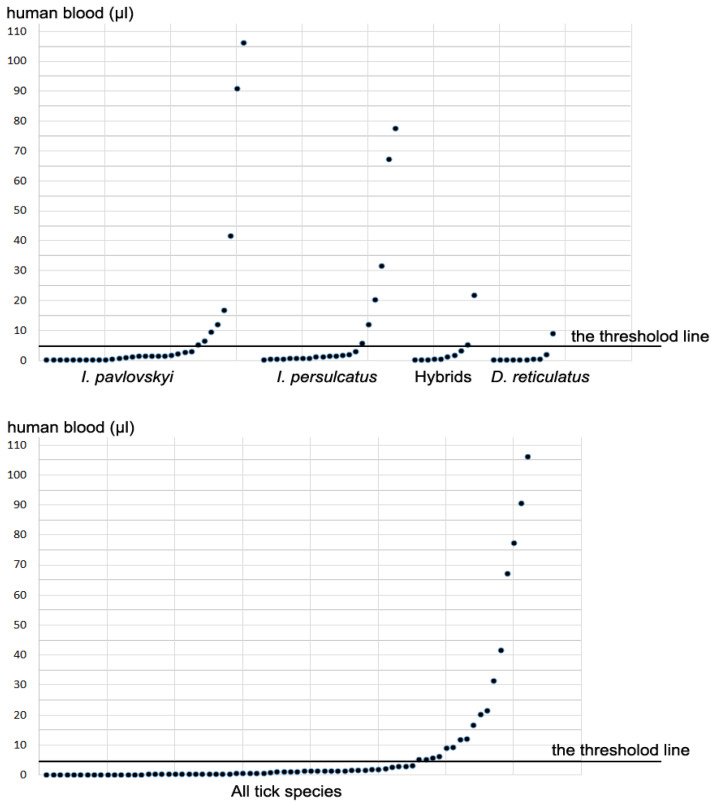
The distribution of PCR-positive ticks with human DNA equivalent to different amounts of blood.

Figure 3 demonstrates the distribution of individuals containing different amounts of human DNA among various tick species. Ticks may have picked up trace amounts of human material while moving across the skin or during their attachment, and it is impossible to distinguish ticks contaminated by human DNA from skin from ticks that have just begun to feed. In this study, ticks containing human DNA in the amount equivalent to >5 µL of blood and based on the distribution of PCR-positive ticks (Figure 3), were considered as “engorged”. Ticks, containing human DNA corresponding to 0.4–5.0 µL of blood, were considered “non-engorged”.

The portion of ticks containing human DNA varied from 17.2% to 36.2% between different tick species, being the lowest for hybrids and the highest for *I. persulcatus* (Table 2); the difference was not significant between any of the tick species, *χ*^2^ (3, *N* = 296) = 6.27, *p* = 0.10. The portion of “engorged” ticks (>5 µL of blood) also varied depending on tick species and constituted 2.3%, 3.4%, 5.8%, and 10.6%, for *Dermacentor* spp., hybrids, *I. pavlovskyi*, and *I. persulcatus*, respectively; however, the difference among different tick species was not significant, *χ*^2^ (3, *N* = 296) = 3.8, *p* = 0.29. Nevertheless, the amount of human blood in a tick varied significantly between tick species according to the results of the Kruskal–Wallis H test, *H* (3) = 8.0035, *p* = 0.046. Further pairwise analysis using the Mann–Whitney U Test showed that the amount of blood detected in *Dermacentor* spp. was significantly lower than in *I. pavlovskyi* (*z* = 2.14, *p* = 0.03) and *I. persulcatus* (*z* = 2.81, *p* = 0.005). No significant difference between other species pairs was detected.

### 3.3. Detection of Borrelia spp. in Ticks

Spirochetes from *B. burgdorferi* s.l. species complex and *B. miyamotoi* were detected in *Ixodes* spp. but not *Dermacentor* spp. ticks. In total, *B. burgdorferi* s.l. was found in 30/296 (10.1%) ticks, including 19/137 (13.9%) *I. pavlovskyi*, 5/58 (8.6%) *I. persulcatus*, and 6/58 (10.3%) hybrids (Table 3). There was no significant difference in the proportion of ticks infected with *Borrelia burgdorferi* s.l. among the different *Ixodes* species, *χ*^2^ (2, *N* = 253) = 1.24, *p* = 0.54. *Borrelia garinii* was the predominant species in all *Ixodes* species, being found in 17/137 (12.4%) *I. pavlovskyi*, 4/58 (6.9%) *I. persulcatus*, and 5/58 (8.6%) hybrids. *B. afzelii* was found only in two *I. pavlovskyi*, whereas *B. bavariensis* was detected in one *I. persulcatus* and a hybrid tick. *I. pavlovskyi* ticks were significantly more often infected with *B. garinii* than other *B. burgdorferi* s.l. genospecies, *χ*^2^ (1, *N* = 19) = 11.84, *p* < 0.001. The association between *I. pavlovskyi* and *B. garinii* was considered strong, with a Cramer’s V of 0.79 (*df* = 1). The difference in the pathogen prevalence among other tick species or between different tick species for the same pathogen was not significant (*p* > 0.5).

The identified *B. burgdorferi* s.l. samples were genetically characterized by the *clp*A gene. A total of twenty-three *B. garinii*, two *B. bavariensis,* and one *B. afzelii clp*A gene fragments with lengths of 719–785 bp were successfully sequenced. Two *B. bavariensis clp*A sequences exactly matched *clp*A alleles 56 and 72 from the PubMLST database, which were common for Russian Siberia and Asian countries. The only determined *clp*A sequence of *B. afzelii* corresponded to allele 36 from the PubMLST database, which also is typical for ticks from Siberia.

**Figure 4:**
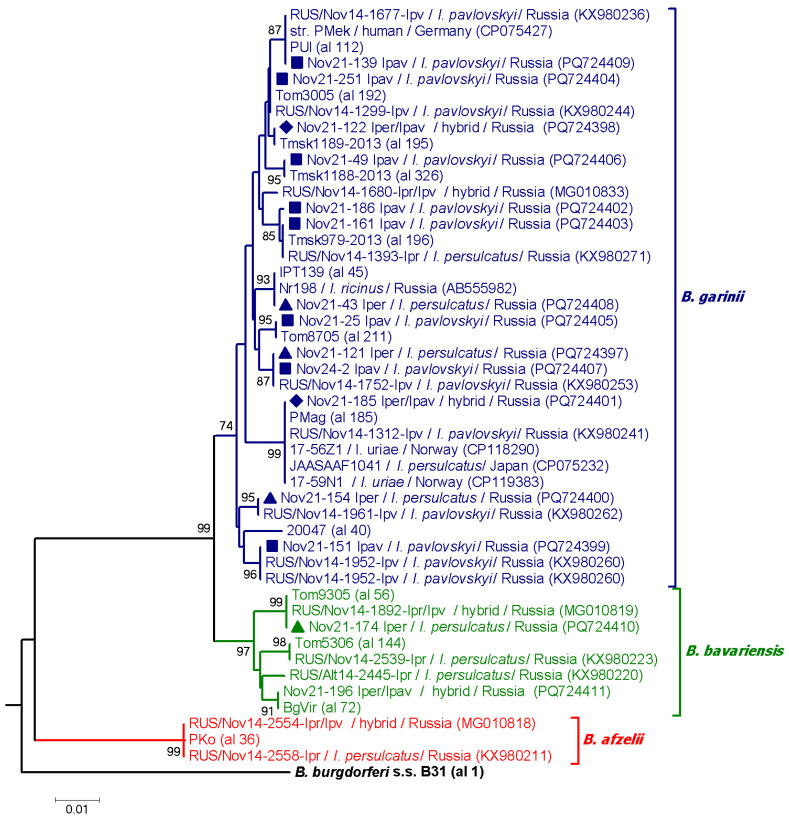
Phylogenetic tree constructed by the ML method (substitution model: Tamura 3-parameter with Gamma distribution (T92 + G)) based on nucleotide sequences of 579 bp fragment of *clp*A gene of *Borrelia* spp. The scale bar indicates an evolutionary distance of 0.01 nucleotide per position in the sequence. Significant bootstrapping values (>70%) are shown on the nodes. *Borrelia burgdrferi s.s.* was used as an outgroup. Legend: ■—*Ixodes pavlovskyi*; ▲—*Ixodes persulcatus*; ♦—hybrids.

Six *B. garinii* sequences contained polymorphic sites and were excluded from further analysis. Among the remaining 17 *B. garinii* sequences, 12 different sequence variants were identified. The sequences from seven ticks exactly matched *clp*A alleles from the PubMLST database (192, 195, 196, 211, and 326), which were previously found only in Western Siberia. The sequences from five ticks were identical to three variants of *clp*A gene sequences, previously identified in ticks from the Novosibirsk province (KX980253, KX980226, and KX980260) but differed from known *clp*A alleles. The sequence from one *I. pavlovskyi* (Nov21-186_Ipavl) was novel and differed by one substitution from allele 196, common for Siberia. Two ticks (*I. persulcatus* and *I. pavlovskyi*) carried the *B. garinii* variant corresponding to the *clp*A allele 112, which is widespread in European countries and Western Siberia. In addition, a novel for Siberia *B. garinii* sequence from *I. persulcatus* (Nov21-43_Iper) matched to *clp*A allele 45, which previously was found only in European countries. Another variant unusual for Siberia was detected in one hybrid (Nov21-185_Iper/Ipav); this variant exactly matched the *clp*A allele 185, which was found in *I. ureae* collected from seabirds from Canada and Norway, one *I. persulcatus* in Japan (CP075232) and one *I. pavlovskyi* in Novosibirsk province (KX980241) (Figure 4).

*Borrelia miyamotoi* was found in eleven ticks, including five *I. pavlovskyi*, five *I. persulcatus,* and one hybrid (Table 3). The prevalence of *B. miyamotoi* did not vary significantly among different *Ixodes* species (*χ*^2^ [2, *N* = 253] = 3.67, *p* = 0.16). Based on *p*66 gene sequence analysis, the determined *B. miyamotoi* sequences were identical to each other and to corresponding sequences from *B. miyamotoi* strains isolated from clinical samples, *I. persulcatus*, and wild rodents (CP036914, CP114703, CP004217, etc.), all belonging to the Siberian subtype.

### 3.4. Detection of Rickettsia spp. in Ticks

Rickettsial DNA was detected in 49/296 (16.6%) ticks: 9/137 (6.6%) *I. pavlovskyi*, 26/58 (44.8%) *I. persulcatus*, 3/58 (5.2%) hybrids, 10/38 (26.3%) *D. reticulatus*, and one *D. silvarum* (Table 4). The portion of ticks infected with *Rickettsia* spp. varied significantly between different tick species, *χ*^2^ (3, *N* = 296) = 51.4, *p* < 0.00001. Three rickettsial species were identified. “*Candidatus* R. tarasevichiae” was found in 26/58 (44.8%) *I. persulcatus*, 1/137 (0.7%) *I. pavlovskyi*, and 3/58 (5.2%) hybrids. *Rickettsia raoultii* was detected in 10/38 (26.3%) *D. reticulatus*, 4/137 (2.9%) *I. pavlovskyi*, and one *D. silvarum*, whereas *R. helvetica* was found in 3/137 (2.2%) *I. pavlovskyi*. In addition, *Rickettsia* sp. not belonging to the known species was identified in one *I. pavlovskyi.* Thus, “*Candidatus* R. tarasevichiae” was found significantly more often in *I. persulcatus* than in other tick species (*χ*^2^ [2, *N* = 253] = 79, *p* < 0.0001), while *R. raoultii* was significantly more often detected in *D. reticulatus* than in *I. pavlovskyi* (*χ*^2^ [1, *N* = 175] = 22.1, *p* < 0.00001). In the first case, the association between the pathogen and the tick species was considered strong, with a Cramer’s V of 0.56 (*df* = 2). In the second case, the association was considered moderate, with a Cramer’s V of 0.36 (*df* = 1).

Positive samples were genotyped by the *glt*A and *omp*B genes. The determined “*Candidatus* R. tarasevichiae” sequences from different tick species were identical and matched to the known corresponding sequences of “*Candidatus* R. tarasevichiae” from *I. persulcatus* from Russian Siberia and the Far East (KM288450, OP72685, etc.).

**Figure 5:**
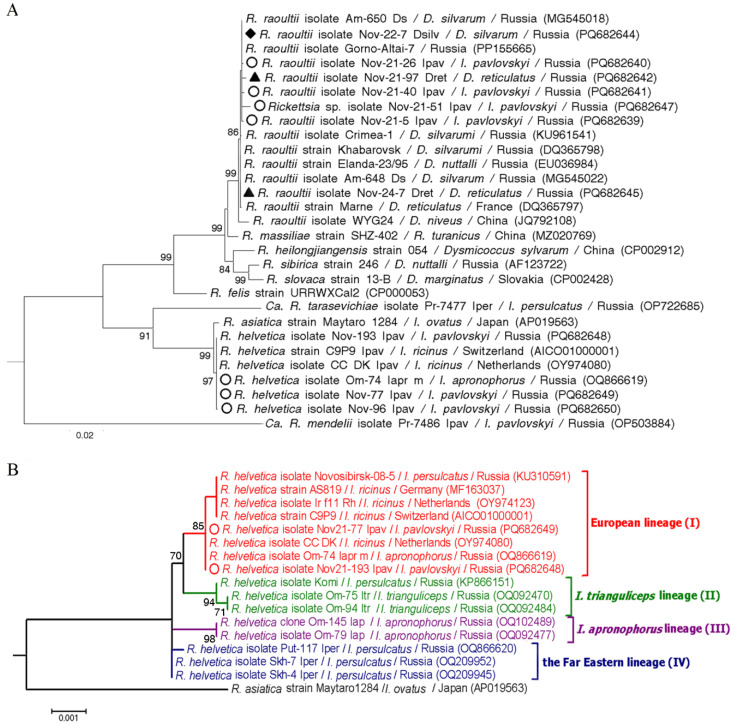
(**A**) Phylogenetic tree constructed by the ML method (substitution model: Tamura 3-parameter with and invariant sites (T92 + I) based on nucleotide sequences of 716 bp fragment of *omp*B gene of *Rickettsia* spp. (**B**) Phylogenetic tree constructed by the ML method (substitution model: Tamura 3-parameter (T92) based on nucleotide sequences of 3097 bp fragment of *omp*B gene of *Rickettsia helvetica*. The scale bars indicate evolutionary distances of 0.02 (**A**) and 0.001 (**B**) nucleotide per position in the sequence. Significant bootstrapping values (>70%) are shown on the nodes. “*Candidatus* Rickettsia mendelii” (**A**) and *Rickettsia asiatica* (**B**) were used as outgroups. Legend: ○—*Ixodes pavlovskyi*; ♦—*Dermacentor silvarum*; ▲—*Dermacentor reticulatus*.

The *glt*A and *omp*B sequences of *R. raoultii* isolate from a single positive *D. silvarum* exactly matched the sequences of *R. raoultii* isolate Am-650_Ds (MG545017, MG545018) previously found in *D. silvarum* from the Russian Far East. The determined *R. raoultii* sequences from eight *D. reticulatus* were identical within both examined genes; the *glt*A gene sequences exactly matched the sequence of the *R. raoultii* strain Marne found in *D. reticulatus* from France (RpA4 genotype, DQ365803) and the *ompB* gene sequences corresponded to the sequence of the *R. raoultii* strain Khabarovsk identified in *D. silvarum* from the Russian Far East (DnS14 genotype, DQ365798). For another two *R. raoultii* isolates from *D. reticulatus* and four isolates from *I. pavlovskyi*, only *ompB* gene sequences were obtained and all these sequences differed from each other. A sequence from *I. pavlovskyi* was identical to that of *R. raoultii* strain Khabarovsk, whereas another five sequences were most similar to the sequence of *R. raoultii* from Western Siberia (isolate Gorno-Altai-7, PP155665), differing from it by 2–3 substitutions (Figure 5A).

For two of three *R. helvetica* isolates, the fragments of *glt*A and *omp*B genes were amplified and sequenced. The obtained sequences of each of the *glt*A and *omp*B genes were identical within the gene and corresponded to sequences of *R. helvetica* from *Ixodes ricinus* from Netherlands (OY974080) and *Ixodes apronophorus* from Russian Siberia (OQ866615 and OQ866619) and differed by one substitution in the *omp*B gene sequence from *R. helvetica* strain C9P9 from *I. ricinus* from Switzerland (NZ_CM001467) (Figure 5B). For the third *R. helvetica* isolate, only an *omp*B gene fragment was amplified; the obtained sequence differed from the other two determined sequences by one substitution.

### 3.5. Detection of Anaplasmataceae Bacteria in Ticks

Three species from the Anaplasmataceae family were found in the examined ticks; however, their prevalence was rather low. The agent of HGA, *Anaplasma phagocytophilum*, was found in three *I. pavlovskyi*; *Ehrlichia muris* was detected in two *I. pavlovskyi*, and *Neoehrlichia mikurensis* was identified in four *I. pavlovskyi* and two *I. persulcatus* (Table 5). Nor *Dermacentor* spp., nor hybrids were infected with bacteria from this family.

### 3.6. Detection of Co-Infections with Bacterial Agents of Ticks

Mixed infections with multiple bacterial agents were observed in ten *Ixodes* spp. ticks, comprising four *I. pavlovskyi*, five *I. persulcatus*, and one hybrid. Eight distinct combinations of mixed infections were identified, with two combinations occurring in two ticks each, and the remaining combinations occurring in single ticks (Table 6). Among co-infected *I. pavlovskyi*, three out of four ticks were infected with *B. garinii* and other agents. In contrast, all co-infected *I. persulcatus* and the hybrid tick contained DNA of “*Candidatus* R. tarasevichiae”. Notably, no *Dermacentor* spp. ticks were found to harbor multiple bacterial infections.

## 4. Discussion

The composition of the tick population in the southern regions of Western Siberia significantly changed in recent decades. In some locations, especially in the suburbs of large cities (Novosibirsk and Tomsk), *I. pavlovskyi* almost completely displaced *I. persulcatus* [9,10]. Moreover, natural *I. persulcatus*/*I. pavlovskyi* hybrids were identified throughout the sympatric areas of *I. persulcatus* and *I. pavlovskyi* [11,12]. The distribution area of *D. reticulatus* ticks also expanded in the last decades and their prevalence near large cities significantly increased [20,24]. The reasons for such rapid changes in the tick population are not entirely clear but may be related to climate change or anthropogenic impact. Thus, global warming can improve the habitat conditions of ticks and significantly affect their hosts, creating more favorable conditions for their spread to new territories. Ongoing forest fragmentation and suburban expansion may also expand tick habitat [33,34]. In Western Siberia, human activity has led to a significant reduction in the number of medium and large mammals, the main hosts of *I. persulcatus* adults, near large cities; on the contrary, the number of birds on which *I. pavlovskyi* adults actively feed is large [1]. Climate warming may also contribute to the expansion of *I. pavlovskyi*. Finally, we cannot exclude that active hybridization events, including backcrossing of hybrids with the parental *I. pavlovskyi* tick, may affect the ability of *I. pavlovskyi* to spread rapidly.

The comparative epidemiological significance of different *Ixodes* spp. remains to be established. Unlike previous investigations [35,36], we genetically identified not only well-known tick species but also *I. persulcatus*/*I. pavlovskyi* hybrids. *Ixodes pavlovskyi* was shown to be the predominant tick species in the vicinity of Novosibirsk, accounting for 46% of ticks attacking humans. The prevalence of other ticks was lower than that amounting to 20% for both *I. persulcatus* and hybrids and only 13% for *D. reticulatus* (Table 2). *Dermacentor marginatus*, *D. nuttalli,* and *D. silvarum* were found in rare cases. Notably, the proportion of *Dermacentor* spp. among ticks attacking residents of Novosibirsk throughout the entire tick activity season may be more significant, since, unlike *Ixodes* spp., ticks of the genus *Dermacento*r have an additional autumn peak of activity in August-September [1,32,35].

The obtained results are in good agreement with the data of a recent study by Kartashov et al. [35], in which *I. pavlovskyi* dominated among ticks attacking humans with a frequency of 43%. Notably, in our previous study, a similar prevalence of *Ixodes* spp. was observed in ticks collected from vegetation; the proportion of *I. pavlovskyi*, *I. persulcatus,* and hybrids was 50%, 17%, and 26%, respectively [12]. This close correspondence was unexpected since questing ticks were collected from vegetation in only five randomly selected locations in the Novosibirsk province, whereas ticks taken from humans could have inhabited anywhere in the region.

The fact that *I. pavlovskyi* and hybrids readily attack humans does not mean that they are able to feed on humans effectively. To study the ability of different tick species to feed on humans, we estimated the amount of human blood in the engorged ticks that were received during feeding. Certainly, this is a relative estimation, since the concentration of DNA in human blood varies from person to person depending on the composition of their blood. Another limitation is the inability to determine whether the small amount of human material was obtained from the skin during tick movement and attachment or it was obtained during blood feeding. To exclude any false positive results, only ticks with significant amounts of human DNA, corresponding to >5 µL of human blood, were considered “engorged”. This amount of human blood corresponds to the weight of at least two unfed *I. persulcatus* females; the average weight of an unfed female was shown to be 2.2 mg [37]. The number of “engorged” ticks was small because in most cases people removed the ticks before or shortly after attachment, and small amounts of human blood are not reliably detectable.

Despite all the above limitations, “engorged” ticks were found among all tick species, with the maximum amount of human DNA corresponding to approximately 106 µL blood for *I. pavlovskyi*, 78 µL for *I. persulcatus*, 22 µL for hybrids, and only 9 µL for *D. reticulatus* (Figure 3). The obtained results first demonstrated that *I. pavlovskyi* and hybrids can effectively feed on humans. This indicates that hybrids, along with *I. persulcatus* and *I. pavlovskyi*, pose a potential danger as carriers of tick-borne infections.

Despite the portion of “engorged” individuals among hybrids being lower compared to *I. pavlovskyi* and *I. persulcatus* (Table 2), the difference was not significant, probably due to an insufficient number of tested ticks. Another limitation of our study is the lack of information on the stage of the examined ticks, which does not allow for a correct comparison of the proportion of “engorged” ticks between different species. Further studies are needed to clarify this point. If the observed trend is confirmed, it would mean that hybrids are less adapted to feeding on humans than their parental species, perhaps because they need more time for attachment. The low number of engorged *Dermacentor* spp. is likely due to the larger size of these ticks, which allows people to notice them.

Ticks that attacked humans were infected with a variety of bacterial agents, including *Borreli*a spp., *Rickettsia* spp., and Anaplasmataceae bacteria. In some cases, mixed infections with multiple pathogens have been identified, meaning that there is a risk of humans becoming infected with multiple pathogens, which may lead to more severe infections.

In Western Siberia, LB and BMD are the most common and severe bacterial tick-borne infections. The main agents of LB in Siberia are *B. afzelii*, *B. bavariensis*, and *B. garinii*; these genospecies were most frequently identified in *Ixodes* spp. ticks and clinical samples [10,38]. Notably, a new species “*Candidatus* Borrelia sibirica” was recently discovered in *Ixodes* spp. in the neighboring Omsk province; however, the pathogenic properties of this species are unknown [39]. Previous studies of ticks collected in various regions of Siberia have demonstrated the association of *I. persulcatus* with *B. afzelii*, *B. bavariensis*, and *I. pavlovskyi* with *B. garinii* [10,39,40]. Unexpectedly, in ticks tested in this study *B. garinii* almost completely displaced *B. afzelii* and *B. bavariensis* and was dominant not only in *I. pavlovskyi* but also in hybrids and *I. persulcatus* (Table 3). This discrepancy may be due to the significant dominance of *I. pavlovskyi* in the tick population and the ability of *I. pavlovskyi* to transmit *B. garinii* to other *Ixodes* species via infected small mammals or by co-feeding.

*Borrelia garinii* is a genetically variable species associated with terrestrial and marine birds, whereas *B. afzelii* and *B. bavariensis* are associated with small mammals. Because of host specificity, *B. garinii* can be transmitted over long distances, and different *B. garinii* genovariants do not cluster by geography or tick species [17]. Since the analyzed ticks could attack humans anywhere, we expected to find new *Borrelia* genovariants.

Indeed, we found one novel *B. garinii* variant and two variants, corresponding to the *clp*A alleles, which were widespread only in the *I. ricinis* distributive area in Europe (allele 45) or in both European countries and Western Siberia (allele 112). Another unusual Siberia variant exactly matched the *clp*A allele 185, closely associated with *I. ureae* and seabirds from Canada and Norway [41]. Despite the close association with marine birds, several *B. garinii* isolates, containing the *clp*A allele 185, were found in single *I. persulcatus*, *I. pavlovskyi*, and *I. persulcatus*/*I. pavlovskyi* hybrid in Japan and Novosibirsk province (ref. [12] and this study). These findings clearly demonstrate the adaptation of the specialized *B. garinii* variant to a broader host range.

In this study, *B. miyamotoi*, a spirochete of the relapsing fever group, was detected in all *Ixodes* spp., including hybrids, with a prevalence of 4.3% among *Ixodes* spp. (Table 3). The observed prevalence of ticks attacking humans was consistent with the *B. miyamotoi* prevalence of ticks collected from vegetation in the Novosibirsk province, which ranged from 3.9% to 6.7% for various *Ixodes* species [12,27]. The stable and relatively high prevalence of *B. miyamotoi* in ticks explains the consistently high incidence of BMD in Novosibirsk province, which is only twice as rare as LB and accounts for 10% of hospitalized patients [42].

In Western Siberia, rickettsioses can be caused by several *Rickettsia* spp.; most cases were caused by *R. sibirica*, followed by *R. raoultii*. In rare cases, “*Candidatus* R. tarasevichiae”, *Rickettsia aeschlimannii*, and *Rickettsia slovaca* were recorded as causative agents of infections in Novosibirsk province [29]. For *Rickettsia* spp., the main route of transmission is transovarial; thus, their association with certain tick species should be more specific compared to *B. burgdorferi* s.l. The study of ticks taken from humans demonstrated a close association of *I. persulcatus* with “*Candidatus* R. tarasevichiae” and *D. reticulatus* with *R. raoultii* (Table 3); a similar association was shown for questing ticks collected from various locations in Western Siberia [10,12,22]. Despite the high prevalence of “*Candidatus* R. tarasevichiae” and *R. raoultii* in ticks removed from humans, cases of infections with these pathogens are quite rare, which can be explained by the low pathogenicity of these agents.

Both *R. raoultii* and *R. helvetica* are genetically variable species. This study demonstrated higher genetic variability of *R. raoultii* samples obtained from *I. pavlovskyi* compared to samples from *D. reticulatus*. These results correspond to our previous findings that *R. raoultii* isolates from *Ixodes* spp. ticks are more variable than isolates from *Dermacentor* spp. [10,12,22]. Notably, *Ixodes* spp. ticks infected with *R. raoultii* were taken from humans, consistent with the high genetic variability of *R. raoultii* in clinical samples [29].

It has recently been shown that *R. helvetica* isolates are reliably subdivided into four genetic lineages [26]. The European lineage is the most numerous and includes all genotyped *R. helvetica* isolates from *I. ricinus* from European countries [43] and from *I. persulcatus* from Western Siberia [26,35]. In this study, *R. helvetica* isolates from *I. pavlovskyi* were first genotyped by a long fragment of the *omp*B gene, which showed that these isolates also belong to the European lineage. Despite the presence of pathogenic *R. helvetica* in *Ixodes* spp. ticks, no cases of *R. helvetica* infection have been registered in Novosibirsk province. This may be due to the fact that *R. helvetica* infection has symptoms atypical for rickettsioses [44,45].

*Rickettsia sibirica*, which is the causative agent of widespread STT, was not detected among the examined ticks. The main vectors of *R. sibirica* are *D. nuttalli*, *D. silvarum*, and *D. marginatus* [46,47], which, as shown in this and other studies [35], rarely attack humans in the vicinity of Novosibirsk. Due to the high pathogenicity of *R. sibirica*, even rare cases of human infection with this agent can manifest as severe infection.

Three potentially pathogenic members of the Anaplasmataceae family, *A. phagocytophilum*, *E. muris,* and *N. mikurensis*, were found in ticks attacking humans; the prevalence of each species was low and did not exceed 2%. There are no confirmed cases of anaplasmosis and ehrlichiosis in humans in Siberia [38], so the epidemiological significance of the identified Anaplasmataceae bacteria is probably minor.

In conclusion, the obtained results indicated that *I. pavlovskyi* currently has the greatest epidemic significance for residents of Novosibirsk; these ticks attack humans more than 2–3 times as often as *I. persulcatus*, hybrids, and *D. reticulatus* and are able to effectively feed on humans. It was first shown that *I. persulcatus*/*I. pavlovskyi* hybrids can readily attack humans and feed on them effectively. Ticks attacking humans were infected with three genospecies of *B. burgdorferi* s.l. species complex, *B. miyamotoi* from the relapsing fever group, three species of *Rickettsia*, and three species from the Anaplasmataceae family. Notably, *B. garinii* almost completely displaced *B. afzelii* and *B. bavariensis* from the tick population. The obtained results indicate a high risk of infection in humans with causative agents of LB, primarily *B. garinii*.

## Figures and Tables

**Table 2:** Human DNA in different tick species determined by RT-PCR.

Amount (µL) of Human Blood in a Tick	No (%) of Ticks Containing Different Amount of Human Blood				
	*I. pavlovskyi* (n = 137)	*I. persulcatus* (n = 58)	Hybrids (n = 58)	*Dermacentor* spp. (n = 43)	All species (n = 296)
**Nd (<0.4)**	**106 (77.4)**	**37 (63.8)**	**48 (82.8)**	**33 (76.7)**	**224 (75.7)**
0.4–0.9	12	7	5	8	32
1.0–3.0	9	7	2	1	19
3.0–5.0	2	1	1	0	4
**“non-engorged” (0.4–5.0)**	**23 (16.8)**	**15 (25.9)**	**8 (13.8)**	**9 (20.9)**	**55 (18.6)**
5.0–10	3	1	1	1	6
10–50	3	3	1	0	7
50–106	2	2	0	0	4
**“engorged” (>5)**	**8 (5.8)**	**6 (10.3)**	**2 (3.4)**	**1 (2.3)**	**17 (5.7)**
**Total positive (0.4–106)**	**31 (22.6)**	**21 (36.2)**	**10 (17.2)**	**10 (23.3)**	**72 (24.3)**

**Table 3:** Prevalence of *Borrelia* spp. in Ixodidae ticks collected from humans.

Tick Species	No. of Ticks	No. (%) of Ticks Containing DNA of Tested Agents *				
		Bg	Ba	Bb	*B. burgdorferi* s.l.	Bm
*I. pavlovskyi*	137	17 (12.4)	2 (1.5)	0	19 (13.9)	5 (3.6)
*I. persulcatus*	58	4 (6.9)	0	1 (1.7)	5 (8.6)	5 (8.6)
Hybrids	58	5 (8.6)	0	1 (1.7)	6 (10.3)	1 (1.7)
*Dermacentor* spp.	43	0	0	0	0	0
**All species**	**296**	**26 (8.8)**	**2 (0.7)**	**2 (0.7)**	**30 (10.1)**	**11 (3.7)**

Abbreviations: Bg—*Borrelia garinii*; Ba—*Borrelia afzelii*; Bb—*Borrelia bavariensis*; Bm—*Borrelia miyamotoi*. * Including cases of mixed infection.

**Table 4:** Prevalence of *Rickettsia* spp. in Ixodidae ticks collected from humans.

Tick Species	No. of Ticks	No. (%) of Ticks Containing DNA of tested Agents				
		Rt	Rr	Rh	Rsp	Total *Rickettsia* spp.
*I. pavlovskyi*	137	1 (0.7)	4 (2.9)	3 (2.2)	1 (0.7)	9 (6.6)
*I. persulcatus*	58	26 (44.8)	0	0	0	26 (44.8)
Hybrids	58	3 (5.2)	0	0	0	3 (5.2)
*D. reticulatus*	38	0	10 (26.3)	0	0	10 (26.3)
*D. nuttalli*/*D. silvarum*	3	0	1	0	0	1
*D. marginatus*	2	0	0	0	0	0
**All species**	**296**	**30 (10.1)**	**15 (5.1)**	**3 (1.0)**	**1 (0.3)**	**49 (16.6)**

Abbreviations: Rt—“*Candidatus* Rickettsia tarasevichiae”; Rr—*Ricketsia raoultii*; Rh—*Rickettsia helvetica*; Rsp—*Rickettsia* sp.

**Table 5:** Prevalence of Anaplasmataceae bacteria in Ixodidae ticks collected from humans.

Tick Species	No. of Ticks	No. (%) of Ticks Containing DNA of Tested Agents			
		Aph	Em	Nm	Total Anaplasmataceae
*I. pavlovskyi*	137	3 (2.2)	2 (1.5)	4 (2.9)	9 (6.6)
*I. persulcatus*	58	0	0	2 (3.4)	2 (3.4)
Hybrids	58	0	0	0	0
*Dermacentor* spp.	43	0	0	0	0
**All species**	**296**	**3 (1.0)**	**2 (0.7)**	**6 (2.0)**	**11 (3.7)**

Abbreviations: Aph—Anaplasma phagocytophilum; Em—Ehrlichia muris; Nm—Neoehrlichia mikurensis.

**Table 6:** Prevalence of co-infections with different bacterial agents in Ixodidae ticks collected from humans.

Variants of Co-Infections	No (%) of Ticks of Different Species Containing DNA of Two Agents				
	*I. pavlovskyi* (n = 137)	*I. persulcatus* (n = 58)	Hybrids (n = 58)	*Dermacentor* spp. (n = 43)	Total Ticks (n = 296)
*B. garinii* + *B. miyamotoi*	1	0	0	0	1
*B. garinii + R. helvetica*	1	0	0	0	1
*B. garinii +* Ca. R. tarasevichiae	0	1	0	0	1
*B. bavariensis +* Ca. R. tarasevichiae	0	1	1	0	2
*B. garinii + E. muris*	1	0	0	0	1
*B. miyamotoi +* Ca. R. tarasevichiae	0	2	0	0	2
*B. miyamotoi + N. mikurensis*	1	0	0	0	1
Ca. R. tarasevichiae + *N. mikurensis*	0	1	0	0	1
**All variants of co-infections**	**4 (2.9)**	**5 (8.6)**	**1 (1.7)**	**0**	**10 (3.4)**

Abbreviations: Ca. R. tarasevichiae—“*Candidatus* Rickettsia tarasevichiae”.

## Data Availability

The data are contained within the article.

## Supplementary Materials

The following supporting information can be downloaded at: https://www.mdpi.com/article/10.3390/pathogens14040315/s1, Figure S1: The linear dependence between the dilution degree and the threshold cycle of the control human DNA; Figure S2: Amplification curve for the control human DNA and tested ticks.
