# Supplementary material for: Spectrum of Ixodidae Ticks Attacking Humans in Novosibirsk Province, Russian Siberia, and Their Association with Tick-Borne Bacterial Agents
Source: Pathogens. 2025 Mar 25;14(4):315. doi: 10.3390/pathogens14040315 (PMC12030029; doi:10.3390/pathogens14040315)
Supplement: Supplementary file 1 [file pathogens-14-00315-s001.zip › pathogens-3521761-supplementary.pdf]

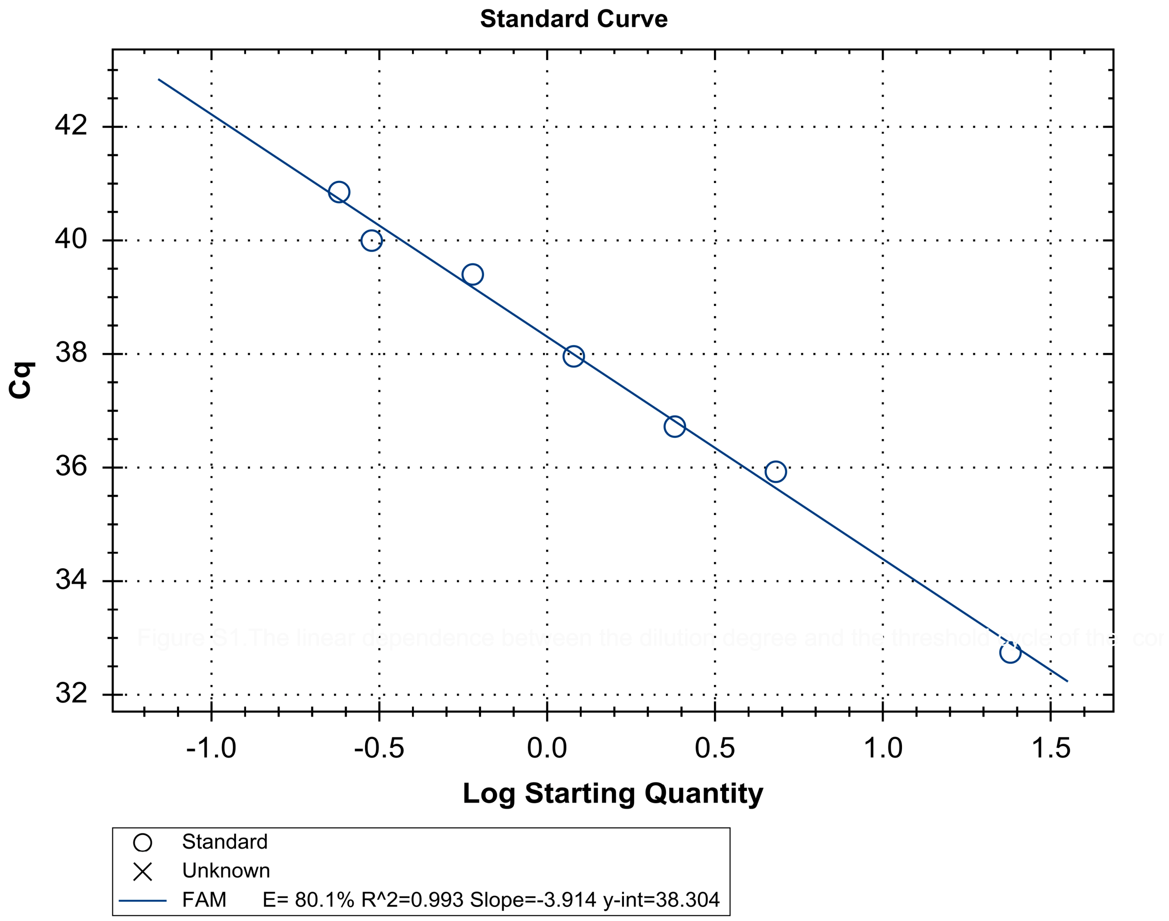

Figure S1.The linear dependence between the dilution degree and the threshold cycle of the control human DNA. The Y-axis shows the number of cycles.

The X-axis shows the quantity of control human DNA equivalent to the different volume of human blood.

The circles correspond to 0.24, 1.2, 2.4, 4.8, 9.6, 19.2, and 24 μl of human blood.

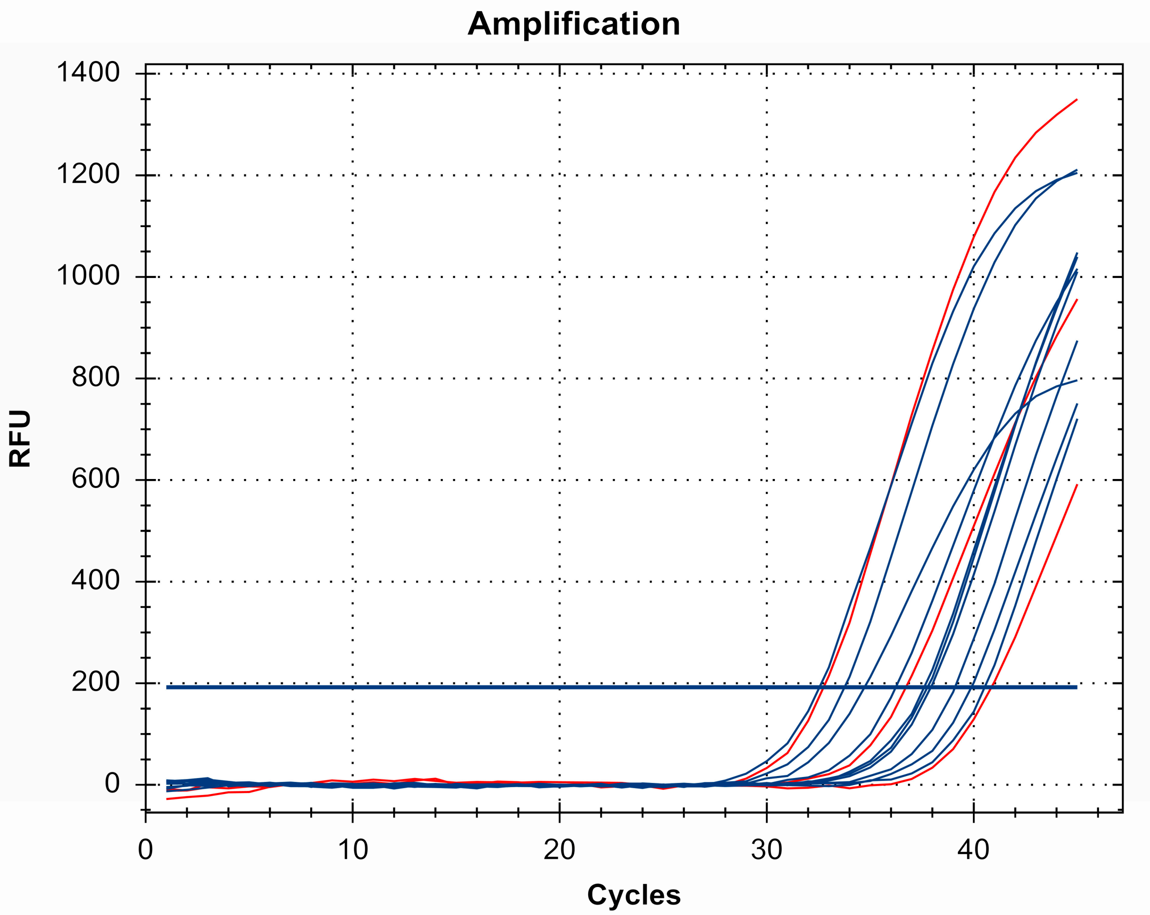

Figure S2. Amplification curve for the control human DNA and tested ticks.
Red curves - control human DNA taken in amounts equivalent to 0.24, 2.4, and 24 µl of human blood.
Blue curves - DNA from randomly selected tested ticks.
